# Cells with stemness features are generated from *in vitro* transformed human fibroblasts

**DOI:** 10.1038/s41598-018-32197-5

**Published:** 2018-09-14

**Authors:** Bartolo Bono, Paola Ostano, Martina Peritore, Ilaria Gregnanin, Cristina Belgiovine, Manuela Liguori, Paola Allavena, Giovanna Chiorino, Ilaria Chiodi, Chiara Mondello

**Affiliations:** 1 Istituto di Genetica Molecolare, CNR, Via Abbiategrasso, 207, 27100 Pavia, Italy; 2 Dipartimento di Biologia e Biotecnologie, Pavia University, via Ferrata 9, 27100 Pavia, Italy; 3 “Cancer Genomics Laboratory” Fondazione Edo ed Elvo Tempia Valenta, Via Malta, 3, 13900 Biella, Italy; 4 Department of Biomedical Sciences, Humanitas University, Via Rita Levi Montalcini, 20090, Pieve Emanuele, Milan, Italy; 5 IRCCS Humanitas Clinical and Research Center, 20089 Rozzano, Milan, Italy

## Abstract

Cancer stem cells (CSCs) have been involved in the maintenance, progression and relapse of several tumors, but their origin is still elusive. Here, *in vitro* transformed human fibroblasts (cen3tel cells) and the tumorsphere assay were used to search for and possibly characterize CSCs in transformed somatic cells. Cen3tel cells formed spheres showing self-renewal capacity and Sox2 overexpression, suggesting that they contained a subset of cells with CSC-like features. Sphere cells displayed deregulation of a c-*MYC*/miR-34a circuitry, likely associated with cell protection from apoptosis. Gene expression profiles of sphere cells revealed an extensive transcriptional reprogramming. Genes up-regulated in tumorspheres identified processes related to tumorigenesis and stemness, as cholesterol biosynthesis, apoptosis suppression, interferon and cytokine mediated signalling pathways. Sphere cells engrafted into NSG mice more rapidly than adherent cells, but both cell populations were tumorigenic. These results indicate that, during transformation, human somatic cells can acquire CSC properties, confirming the high plasticity of tumor cells. However, CSC-like cells are not the only tumorigenic population in transformed cells, indicating that the CSC phenotype and tumorigenicity can be uncoupled.

## Introduction

Tumors are highly heterogeneous entities, composed of cells with different genetic and epigenetic features and different phenotypes. Tumor cell heterogeneity is a major problem in tumor treatment^1^ and the presence of cells with different tumorigenic capacities and drug resistance can make tumor eradication difficult.

In several malignancies, a layer of heterogeneity is given by the occurrence of a subset of tumor cells, named cancer stem cells (CSCs), characterized by stemness properties. CSCs are important players in cancer development, progression and recurrence, being endowed with a high tumorigenic potential and elevated resistance to antitumor therapies^2^.

The origin of tumor heterogeneity, and particularly of CSCs, is a matter of debate. A large body of evidence indicates that tumor cells own a high degree of plasticity with a bidirectional interchange between cells with different phenotypes^3^; the genesis of more differentiated cells from CSCs, as well as the origin of CSCs from bulk tumor cells have been reported, indicating that strategies targeting all tumor cell types have to be developed to eradicate tumors^4^.

Cells with a CSC phenotype have also been shown to be generated from human somatic cells transformed *in vitro* by oncogenic insults. The SSEA1 antigen was found to mark CSCs in a fibroblast transformed cell population^5^, while exogenous *GNL3* expression appeared to confer cancer stem cell properties to transformed human fibroblasts or kidney epithelial cells^6^. Similarly, stem cell-like cancer cells were generated by the induction of the epithelial-mesenchymal transition (EMT) in *in vitro* transformed mammary cells^7,8^.

In our laboratory, an *in vitro* model system for human fibroblast transformation was developed from hTERT immortalized fibroblasts^9–14^. Immortalized cells, named cen3tel, spontaneously and gradually underwent neoplastic transformation during culture propagation, becoming able to induce tumors when injected into immunocompromised mice. Once transformed, cells became more and more aggressive with further propagation in culture, as shown by a decrease in the time required to develop tumors in mice; moreover, the most aggressive cells were able to form metastasis when injected into the tail vein of immunocompromised mice^11^. Thus, cells at different stages of propagation after TERT immortalization represent cells at different phases along the way to transformation.

In this work, cen3tel cells were exploited to study the heterogeneity of *in vitro* transformed cell populations and, particularly, the possible presence of cells with the CSC phenotype, with the aim of identifying pathways involved in their genesis and maintenance.

## Results

### Cen3tel cells at advanced stages of transformation form spheres in non-adherent culture conditions

The cen3tel cellular system has been described in the Methods section. To search for CSCs in transformed somatic cells, SSEA1 expression was first checked in cells at the latest stages of tumorigenicity, cen3tel 600 and cen3tel 1000 cells, because this antigen was shown to identify CSCs in *in vitro* transformed fibroblasts^5^, but no positive cells were present in the cell populations (data not shown). An alternative and more general approach was thus used to select for CSCs: the sphere forming technique, which relies on the evidence that cells with stemness features preferentially respond to growth factors and grow in suspension as spherical clusters in the absence of serum^15^.

**Figure 1:**
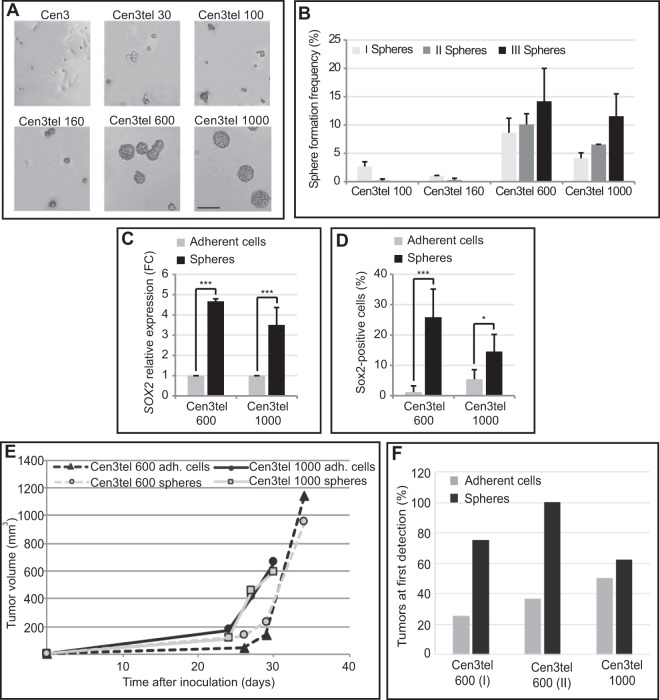
(**A**) Morphology of cen3 primary fibroblasts and cen3tel cells at different stages of propagation (around PDs 30, 100, 160, 600 and 1000) plated in non-adherent culture conditions, in serum free medium supplemented with EGF and FGFb. Cells grown for 7 days in sphere-forming conditions are shown in the pictures taken with a 10X objective. Bars = 200 µm. (**B**) Frequencies of primary, secondary and tertiary spheres from cen3tel cells at different PDs. Frequencies of cen3tel cells at PD 100 and 160 were measured 14 days after cell seeding, while those of cen3tel cells at PD 600 and PD 1000 after 7 days. Mean and standard deviation (error bars) values were calculated from three independent experiments. (**C**) RT-qPCR analysis of *SOX2* expression in cen3tel 600 and 1000 sphere cells. *SOX2* expression in each sphere sample is expressed as fold change (FC) relative to the expression in the corresponding adherent cells. The plot shows the average (FC) of three independent experiments. (**D**) Cytofluorimetric analysis of Sox2 expression showing the percentage of Sox2 positive cells in cen3tel 600 and 1000 sphere cells and adherent cells. Values are the average of the results of three independent experiments. Error bars: standard deviations. ***p < 0.005, *p < 0.05. (**E**,**F**) Tumor induction by adherent and sphere cells in NSG mice. (**E**) Tumor growth; (**F**) percentage of tumors found at the first time of detection (26 days for cen3tel 600 cells and 21 days for cen3tel 1000. Cen3tel 600 (I), first experiment, 4 inoculi; cen3tel 600 (II), second experiment, 8 inoculi; cen3tel 1000, 8 inoculi).

The analysis of sphere formation in cen3tel cells at different stages of propagation revealed that tumorigenic cen3tel 600 and 1000 were indeed able to form spheres when plated in the absence of serum and in the presence of growth factors (Fig. 1A). Moreover, sphere cells were able to form spheres with increasing frequency when replated in sphere forming conditions for successive passages, indicating that they were endowed with self-renewal capacity (Fig. 1B). In contrast, primary cen3 fibroblasts and non-transformed cen3tel cells (cen3tel 30) did not form spheres (Fig. 1A), while cen3tel cells at the early phases of transformation and tumorigenicity (cen3tel 100 and 160, respectively) formed very small spheres at a low frequency and sphere-derived cells did not show self-renewal capacity (Fig. 1A,B). This suggests that the ability to grow in spheres is not simply associated with the tumorigenic phenotype, but requires the acquisition of specific features during the progression of the transformation process. Hereafter, all the results reported will concern cen3tel 600 and 1000 cells.

Spheres obtained from cen3tel 600 and 1000 cells derived from single cells. In fact, plating single cen3tel 600 or cen3tel 1000 cells in sphere forming conditions (see the Methods section) spheres were obtained (frequency around 15% for single cen3tel 600 cells and 3% for single cen3tel 1000 cells). The frequency of sphere formation was higher in cen3tel 600 cells than in cen3tel 1000 cells, while sphere dimension was greater in the latter. After 7 days of growth, the average number of cells *per* sphere was about 100 and 125 in cen3tel 600 spheres and cen3tel 1000 spheres, respectively. Plating single sphere-derived cells, the frequency of sphere formation increased up to about 60–70% in both cell lines, confirming that spheres were enriched in sphere forming cells.

To test whether cells with the ability to grow in spheres represented a defined stable subpopulation of tumorigenic cen3tel cells, sphere formation was analysed in clonal populations derived from cen3tel 600 cells. Given that adherently growing cells formed spheres with a frequency around 10%, it could be envisaged that, if only cells with specific characteristics bore the capacity to grow in spheres, only a small proportion of clonal populations, those originated from these cells, should be able to form spheres. Sphere formation was analysed in 8 clones, each derived from a single cen3tel cell propagated for about 22–23 PDs. All the clones formed spheres, albeit with variable frequency (Table S1). This result makes the possibility that sphere formation capacity is a stable and intrinsic property of some cen3tel cells unlikely, while it suggests that sphere formation capacity defines a cellular state that can be acquired by tumor cells within the population.

The analysis of the expression of the stemness regulator genes *SOX2*, *POU5F1* (encoding for Oct-4) and *NANOG*^16^ revealed that the SOX2 mRNA level was significantly higher in cen3tel 600 and 1000 sphere cells compared to their adherently growing counterparts (Fig. 1C), while no significant differences were observed for *POU5F1* and *NANOG* expression (data not shown).

*SOX2* expression was then tested at single cell level by cytofluorimetric analysis. Cells grown in adhesion contained a small percentage of Sox2 positive cells, which increased in sphere cells (Fig. 1D). The percentage of cells positive to Sox2 was quite variable among experiments, probably because Sox2 positive and negative cells could be generated with different frequencies during sphere growth.

The tumorigenic potential of cen3tel 600 and cen3tel 1000 adherent and sphere cells was analysed inoculating 2.5 × 10^4^ cells bilaterally into the leg muscle of NSG mice and following the development of the tumors. As shown in Fig. 1E, tumor growth was similar for adherent and sphere cells for both cen3tel cells. However, tumors induced by sphere cells, especially by cen3tel 600 cells, tended to appear earlier than those induced by adherent cells (Fig. 1F). Thus, cen3tel sphere cells showed the capacity to engraft more rapidly than adherent cells.

### c-*MYC*, *GNL3*, *NOTCH1* and miR-34a expression is co-ordinately and reversibly deregulated in sphere cells

**Figure 2:**
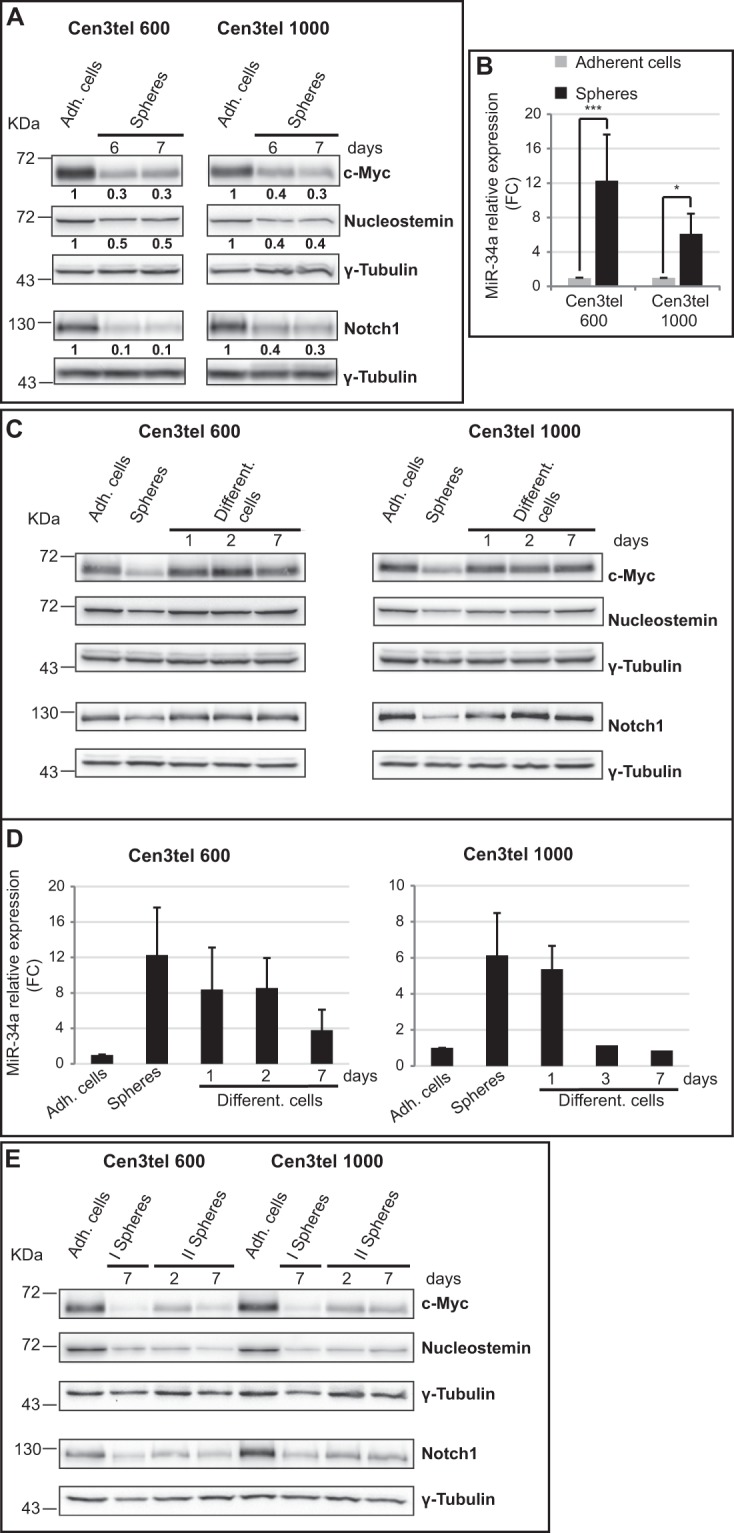
c-Myc, Nucleostemin, Notch1 and miR-34a expression in cen3tel 600 and 1000 sphere cells. Sphere cells were collected at 6 or 7 days of growth in serum free medium and the expression levels of c-Myc, Nucleostemin and Notch1 (**A**) were analyzed by western blotting; miR-34a expression (**B**) was analyzed in 6 day sphere by RT-qPCR. (**A**) For each protein, the relative intensity of the band in each sphere sample respect to the corresponding adherent cells is reported in bold below the corresponding lane. (**B**) MiR-34a expression in each sphere sample is indicated as FC relative to the expression in the corresponding adherent cells. Values are the mean of the results of three independent experiments. Error bars: standard deviations. *p < 0.05, ***p < 0.005 (**C**) Analysis by western blotting of c-Myc, Nucleostemin and Notch1 expression and (**D**) of miR-34a by RT-qPCR in sphere cells allowed differentiating in serum containing medium for different time intervals (indicated as Different. cells). In (**D**) miR-34a expression levels in sphere cells are indicated as FC relative to the corresponding adherent cells and are the mean of the results of three independent experiments. Error bars: standard deviations. (**E**) Analyisis of c-Myc, Nucleostemin and Notch1 expression in cen3tel 600 and 1000 primary spheres (I Spheres) and secondary spheres (II Spheres) at different days of growth in sphere-forming medium. Secondary spheres were obtained by re-plating I sphere cells in sphere-growing medium. For all the western blots, γ-Tubulin was used as control for protein loading.

To further characterize the molecular features of cells growing in spheres, the expression of genes known to be linked to stemness, such as c*-MYC*, *GNL3*, which encodes for nucleostemin, and *NOTCH1*^6,17–19^, was analysed in sphere cells collected at 6 and 7 days of growth. Surprisingly, the level of the three proteins was lower in sphere cells compared to adherently growing cells (Fig. 2A).

Given that c-Myc expression decreases when cells stop proliferating^20^, sphere cell growth was analysed. At different time points (day 5, 6, 7 and 8), spheres were collected and disaggregated and the total number of cells was determined. As shown in Table 1, the number of cells increased between 1.5 and 2 times *per* day during sphere culture, indicating that sphere cells were still proliferating despite the low c-Myc levels.

**Table 1:** Sphere cell number increases during culture.

	Ratio between the number of cells in spheres at different time points		
	d6-d5	d7-d6	d8-d7
cen3tel 600	1.7	1.5	1.5
cen3tel 1000	2.1	1.6	1.9

The expression of miR-34a was then analysed, because this miRNA has a cross talk expression regulation with c*-MYC* and *NOTCH1* and is often down-regulated in cancer stem cells^21–23^. RT-qPCR analysis showed that it was significantly overexpressed in sphere cells compared to adherently growing cells (Fig. 2B).

The expression modulation of these genes was reversible once sphere cells were plated in serum containing medium. Already one day after plating in the presence of serum, the expression levels of c-Myc, nucleostemin and Notch1 regained values comparable to those found in control adherent cells (Fig. 2C). MiR-34a levels also decreased after replating sphere cells in adherent culture conditions, but with a slower kinetics (Fig. 2D).

In contrast, when spheres were disaggregated and sphere cells replated in non-adherent culture conditions, the levels of c-Myc, nucleostemin and Notch1 remained comparable to that observed in primary spheres (Fig. 2E), suggesting that this deregulation was linked to cell growth in suspension. Moreover, it is worthwhile noticing that the expression of these genes appeared to be concerted, since all the genes were regulated in the same way in the different culture conditions.

### Sphere cells do not undergo apoptosis

**Figure 3:**
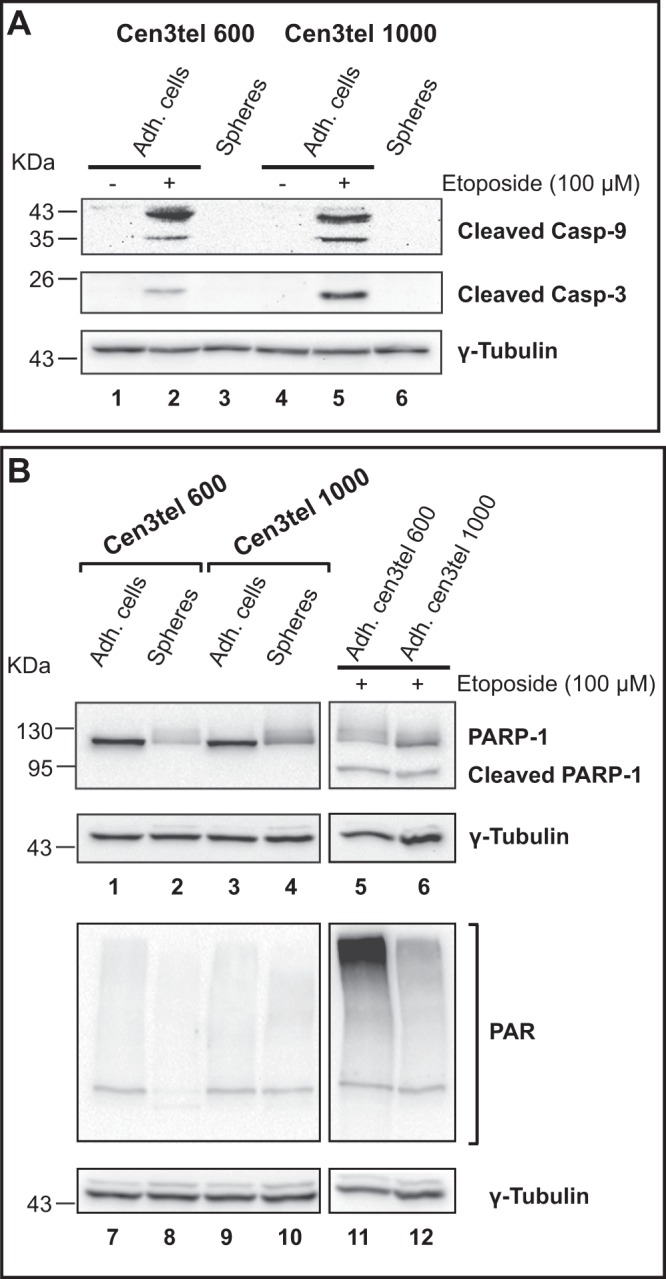
Western blot analysis of apoptotic markers in cen3tel 600 and 1000 sphere and adherent cells. Antibodies recognizing the cleaved active forms of caspases 9 and 3 (**A**) were used, as well as an antibody recognizing both the full-length and the apoptotic cleaved fragment of PARP-1 and an antibody anti-poly ADP ribose (PAR) (**B**). As apoptosis control, adherent cen3tel cells treated with 100 μM etoposide for 24 hours were used. γ-Tubulin was used as loading control.

It is well known that c-Myc promotes apoptosis^24^, therefore decreased c-Myc levels in sphere cells could be functional to protect cells from death, which could occur because of the peculiar structure of spheres, in which cells grow in a highly compact way. In sphere cells, there were no detectable levels of the apoptotic markers cleaved caspase 9 and 3 and the proteolytic PARP-1 fragment, which were present in etoposide treated cells (Fig. 3A). Thus, in sphere cells there is no evidence of apoptosis activation and c-Myc down-regulation could contribute to restrain this process.

Both in sphere cells and etoposide treated cells, PARP1 analysis revealed the presence of a series of bands (Fig. 3B), which probably correspond to poly-ADP-ribosylated PARP-1, suggesting that in sphere cells a stimulus, still to be defined, activates the protein. However, analysing the global levels of protein poly-ADP-ribosylation, a similar extent of modifications was observed in adherent and sphere cells, and a higher level in etoposide treated cells (Fig. 3B), suggesting that etoposide induces a PARP-1 response different from that observed in sphere cells. Further investigations are required to better understand the possible meaning of PARP-1 modification in sphere cells.

### Genome wide gene expression analysis by microarray

**Figure 4:**
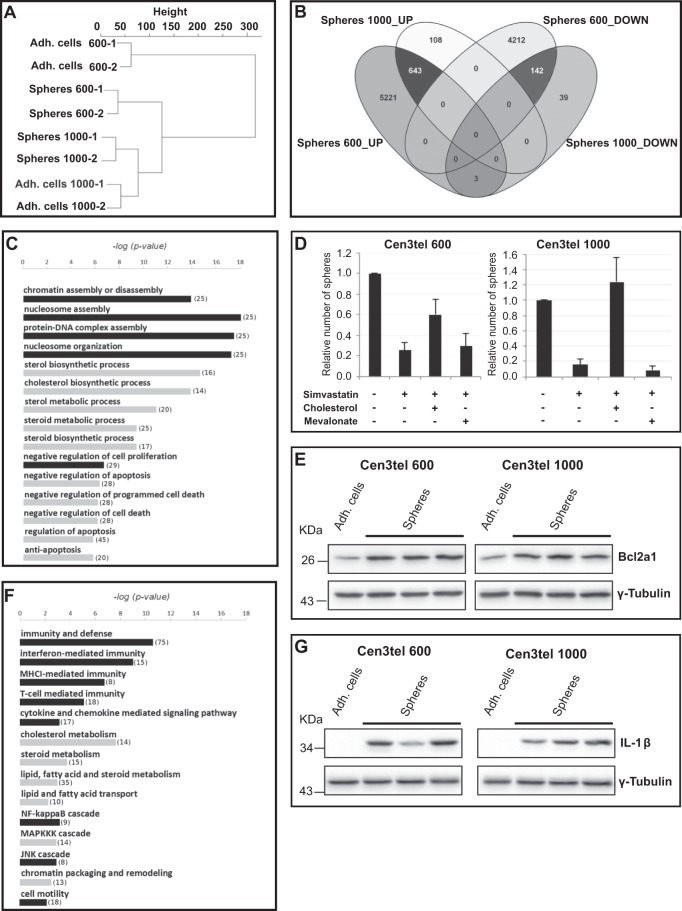
Results and validation of the genome-wide microarray gene expression analysis in cen3tel 600 and 1000 adherent and sphere cells. (**A**) Global gene expression profiling of cen3tel 600 and 1000 adherent and sphere cells. Dendrogram represents the relationship of similarity among the global gene expression profiles of cen3tel 600 and 1000 adherent and sphere cells. The tree diagram was obtained from unsupervised hierarchical clustering using Euclidean distance as similarity metrics and Ward linkage as linkage method. For each cell sample, the two biological replicates are shown. (**B**) Venn diagram representing the genes up- and down-regulated in cen3tel 600 and 1000 sphere cells relatively to their corresponding adherent cells. The black intersections represent the genes commonly deregulated in the two sphere samples. (**C**) GO enrichment analysis by David on the list of the commonly up-regulated genes in cen3tel 600 and 1000 sphere cells. The 15 biological processes with the most significant p-value are represented, terms belonging to the same biological items are clustered and indicated with the same color. Within brackets, the number of deregulated genes falling in each biological process is reported. (**D**) Sphere formation in cen3tel 600 and 1000 cells after inhibition of the cholesterol metabolism gene HMGCR with simvastatin. Cells were plated in sphere forming medium in the presence of simvastatin alone, simvastatin and cholesterol or simvastatin and mevalonate. The number of spheres in exposed cells is relative to that found in untreated cells (**E**) Western blot analysis of Bcl2a1 expression in cen3tel 600 and 1000 adherent and sphere cells. (**F**) GO enrichment analysis using the classification implemented by Panther annotation tool within David on the list of the commonly up-regulated genes in cen3tel 600 and 1000 sphere cells. The 14 biological processes with p-value < 0.01 are represented as described above. (**G**) Western blot analysis of IL1-β expression in cen3tel 600 and 1000 adherent and sphere cells. For all the western blot, γ-Tubulin was used as loading control.

To better investigate sphere cell features, genome wide transcriptomic profiles of cen3tel 600 and cen3tel 1000 sphere cells were compared with those of their adherent counterparts. Global gene expression profiling showed that adherent cen3tel 600 and 1000 cells were separated into two main clusters (Fig. 4A). Cen3tel 1000 sphere cells fell in the same cluster as their adherent counterpart. Cen3tel 600 sphere cells were closer to cen3tel 1000 adherent and sphere cells than to cen3tel 600 adherent cells, indicating that cen3tel 600 sphere cells switched towards a gene expression profile more similar to that of the more aggressive cen3tel 1000 cells.

The number of genes deregulated in cen3tel 600 and 1000 spheres and commonly deregulated in both sphere types are summarized in Fig. 4B. Functional enrichment analyses of the lists of genes commonly up-regulated or down-regulated in cen3tel 600 and 1000 sphere cells were performed (Tables S2) using the David functional annotation tool and the classification implemented by Panther within David (http://david.abcc.ncifcrf.gov/). The results of these analyses are reported in Tables S3 and S4. The study was then focused on the most significant processes associated with the up-regulated genes.

The 15 most significant David BP5 (Fig. 4C) represent 3 major processes. The first 4 terms concern chromatin organization. The genes falling in these terms encode several replication-dependent histone variants and the replication-independent histone variant H2AFJ (Table S5). Deregulation of these genes suggests that a reorganization occurs at the chromatin level in sphere cells, which could in turn have consequences on gene expression.

The second class of processes includes genes related to cholesterol metabolism, mostly to the mevalonate/cholesterol biosynthetic pathway (Table S5). The overexpression of three genes of the pathway, namely *HMGCS1*, *HMGCR*, which encodes the rate limiting enzyme of the pathway, and *MVK* was confirmed by RT-qPCR (Fig. S1A). Blocking the cholesterol biosynthetic pathway by exposing cent3tel cells to the HMGCR inhibitor simvastatin, the frequency of sphere formation decreased (Fig. 4D), indicating that this pathway is actually relevant for this process. Sphere formation was rescued by the concomitant incubation of simvastatin with cholesterol, but not with mevalonate (Fig. 4D).

The third class of processes concerns the regulation of apoptosis, mainly the negative regulation of this pathway, in agreement with evidence reported above that cell death is prevented in sphere cells. The *BCL2A1* gene, whose overexpression is known to have a crucial role in the regulation of the anti-apoptotic response, is among the most up-regulated genes both in cen3tel 600 and 1000 sphere cells; its up-regulation at the protein level was confirmed by western blotting (Fig. 4E).

### Deregulation of cytokine signalling pathway genes in sphere cells

The analysis of the up-regulated genes in sphere cells performed with the Panther tool (Fig. 4F) highlighted the over-representation of 14 processes related not only to cholesterol and steroid metabolism and chromatin organization, but also to immunity and defence, interferon (IFN) mediated immunity, cytokine and chemokine mediated signalling pathway. This suggests a link between sphere formation, inflammation and immune response, all processes know to be related with cancer and stemness. In Table S5, chemokine and interleukin genes overexpressed in sphere cells are reported.

In cen3tel 600 and 1000 spheres, *IL1B* and *IL13RA2* are among the most up-regulated genes (Table S2, Figs S1B, 4F). *IL-6* is also overexpressed both in cen3tel 600 and cen3tel 1000 spheres, albeit at a lower extent (Log_2_FC 0.78 and 1.15, respectively; Table S2).

*IL1B* encodes IL-1β, an inflammatory cytokine that is synthesized as a precursor protein and then cleaved by caspase 1 to generate the secreted active molecule. The IL-1β precursor form was undetectable in adherent cen3tel 600 and 1000 cells, while was overexpressed in sphere cells (Fig. 4G). However, despite the presence of high levels of pro-IL-1β in the cellular protein extracts, IL-1β was not secreted, being undetectable in sphere culture medium (data not shown), suggesting that the protein is not processed to the active form, even if microarray analysis revealed the over-expression of caspase 1, caspase 4 and caspase 5 (Table S2), which are known to be involved in cytokine processing^25^. To our knowledge, nothing is known about a possible function of the endocellular IL-1β precursor protein.

### Deregulation of IFN pathway genes in sphere cells

Out of 168 genes related to the IFN pathway found in the annotation database Amigo (http://amigo.geneontology.org/amigo/search/annotation), 37 genes were significantly overexpressed in cen3tel 600 and 1000 spheres (Table S5).

**Figure 5:**
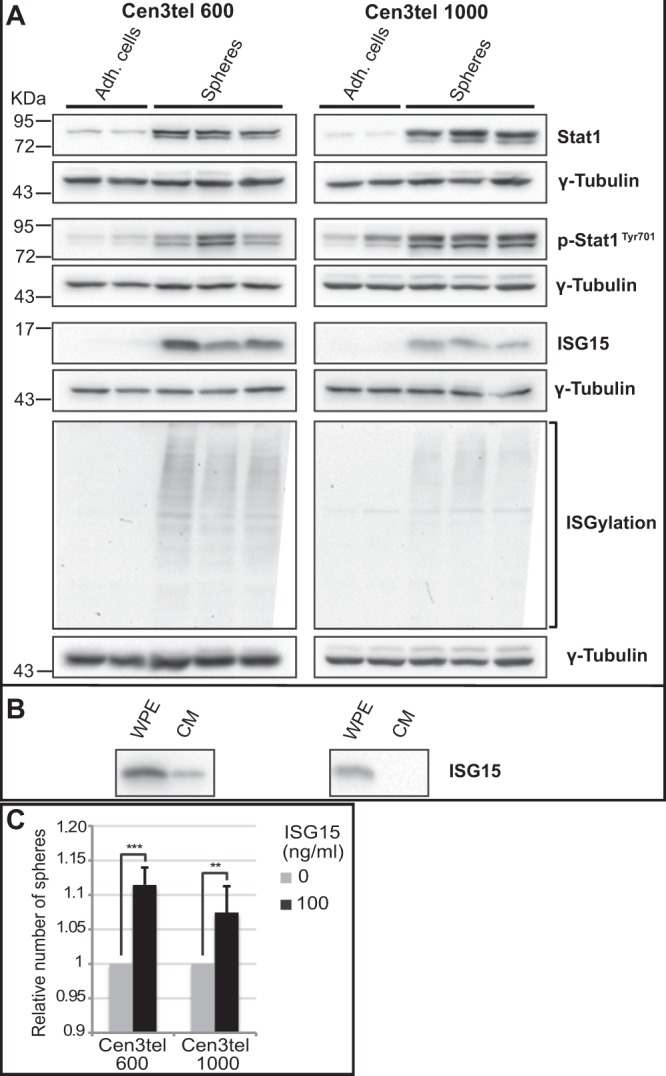
Expression of interferon-related genes in cen3tel 600 and 1000 spheres. (**A**) Western blot analysis of the expression of Stat1, phospho-Stat1, ISG15 and ISGylated proteins in cen3tel 600 and 1000 sphere cells and adherent cells. Phospho-Stat1^Tyr701^ is the active form of the transcription factor Stat1. γ-Tubulin was used as loading control. (**B**) Analysis of the secretion of ISG15 in the culture medium (CM) of cen3tel 600 and 1000 sphere cells. The CM was processed as described in Materials and Methods and analyzed by western blotting with an antibody against ISG15; in the lane of the left, a sample of whole-protein extract (WPE) from sphere cells was loaded as positive control. (**C**) Secreted ISG15 fosters sphere formation in cen3tel 600 and 1000 cells.

Microarray results were confirmed for *IFI6*, *ISG15* and *STAT1* (Figs S1c and 5A). Stat1 controls *ISG15* expression. *ISG15* encodes a small protein that can be covalently linked to several proteins in a process known as intracellular ISGylation, but can also be free in the cytosol or secreted in the extracellular space^26^. Both Stat1 and ISG15 have been found to play a role in tumorigenesis and CSCs^27–30^. Besides Stat1 overexpression, sphere cells also showed high levels of phospho-Stat1^Tyr701^, indicative of its activation (Fig. 5A). ISG15 was barely detectable in adherent cen3tel 600 and 1000 cells while was highly expressed in sphere cells, at a higher extent in cen3tel 600 spheres than in cen3tel 1000 spheres. A similar result was obtained by analysing protein ISGylation, which was much more prominent in sphere cells than in adherent cells and higher in cen3tel 600 spheres than in cen3tel 1000 spheres (Fig. 5A). Thus, in sphere cells there was the activation of the Stat1-ISG15 axis.

ISG15 secretion in culture medium was then tested. As shown in Fig. 5B, a band corresponding to ISG15 was visible in the culture medium of cen3tel 600 sphere cells, but not of cen3tel 1000 cells, probably because it was under the detection limit.

Then, given that ISG15 can foster CSC generation^28^, it was verified whether ISG15 could promote sphere formation in cen3tel 600 and 1000 cells. Cen3tel cells were thus plated in sphere forming medium in the presence of 100 ng/mL of recombinant ISG15 and the number of spheres was counted after 7 days of growth. As shown in Fig. 5C, exogenous ISG15 has a modest but significant positive effect on sphere formation in both cen3tel 600 and 1000 cells.

### Deregulation of cell movement genes in sphere cells

**Figure 6:**
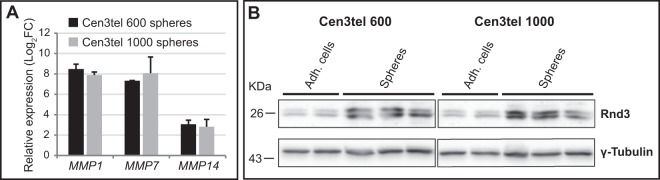
MMP1, MMP7, MMP14 and RND3 expression in cen3tel 600 and 1000 sphere cells. (**A**) Expression of *MMP1*, *MMP7*, *MMP14* by RT-qPCR. In the plots, the expression of each gene in sphere cells is indicated as Log_2_FC relative to its expression in the corresponding adherent cells. Error bars: standard deviations. (**B**) Evaluation of Rnd3 expression levels in cen3tel 600 and 1000 adherent and sphere cells by western blot analysis. γ-Tubulin expression was used as loading control.

During cen3tel transformation, the down-regulation of several metalloproteinase (*MMP*) genes and of the Rho GTPase Rnd3 was found to be associated with a switch from the mesenchymal movement to the amoeboid motility^11^; in fact, tumorigenic cells were characterized by a movement dependent on the activity of the RhoA-dependent kinase ROCK and independent of MMPs^11^. In sphere cells, the expression of *MMP1*, *MMP7* and *MMP14* (Fig. 6A, Table S2) was up-regulated, as well as the expression of *RND3* (Fig. 6B, Table S2), suggesting that cells growing as spheres show a reversion towards a more mesenchymal phenotype compared to adherent cells.

### Gene Set Enrichment Analysis

Running GSEA on the cen3tel sphere up-regulated gene list, a statistically significant enrichment in two genesets of ovarian and breast cancer tumorspheres (geneset “GSE43657”^31^) and in prostate cancer tumorspheres (geneset “GSE10832”^32^) was found. Among the genes commonly up-regulated in these genesets and in cen3tel sphere cells there were genes involved in mevalonate/cholesterol metabolism, chromatin organization and interferon pathway, including *STAT1* and *ISG15*, indicating that the deregulation of these genes is linked to the CSC phenotype.

## Discussion

Tumor cell population heterogeneity and the presence of cells with stemness features is an obstacle to tumor eradication. To study the possible evolution towards a stemness phenotype during cellular transformation of differentiated somatic cells, the cen3tel cellular system and the tumorsphere approach were exploited. The results presented here show that, at the latest stages of transformation, somatic cen3tel cells acquired the capacity to form spheres endowed with stemness features when plated in the absence of serum and in the presence of growth factors. Sphere cells showed the ability to self-renew and overexpressed the stemness transcription factor SOX2. Moreover, cells able to form spheres were constantly generated during *in vitro* propagation of transformed fibroblasts, suggesting that sphere formation capacity is not an intrinsic characteristic of specific cells, but can be stochastically acquired.

These results indicate that somatic transformed cells are highly dynamic entities able to activate peculiar epigenetic programs once they respond to specific growth factors and grow in the absence of solid support. Sphere cells showed a decrease of c-*MYC*, *NOTCH1*, *GNL3* expression, and an increase of miR-34a levels. There is evidence that these molecules are able to cross-regulate their expression^21,22,33,34^. In the adherent-sphere cell system, their regulation was actually concerted; in fact, all of them regained the pattern of expression observed in adherent cells when sphere cells were allowed differentiating in serum containing medium, while maintained the same pattern of expression when sphere cells were plated in sphere forming medium. These observations suggest that these genes are connected in a circuitry and probably act in the same functional process during sphere formation. Myc down-regulation, the decrease in Nucleostemin and Notch levels and the up-regulation of miR-34a could take part in restraining apoptosis in non-adherent growth conditions^35,36^. It can be speculated that *in vivo* this mechanism could contribute to protect cells that detach from the extracellular matrix from apoptosis, allowing them to grow in suspension and disseminate.

Sphere cells showed profound changes in the transcriptional program compared to their adherent counterparts. These changes were greater in cen3tel 600 sphere cells than in cen3tel 1000 sphere cells. During transformation, cen3tel cells displayed a progressive increase in the number of genes deregulated compared to parental fibroblasts and a particularly elevated increase was observed during the transition between cen3tel 600 and cen3tel 1000 cells^13^. Cen3tel 600 sphere global transcription profiling was, actually, more similar to that of cen3tel 1000 adherent and sphere cells than to that of their adherent counterparts, suggesting that sphere cells acquire features linked to more advanced stages of transformation.

A common feature of spheres derived from both cen3tel 600 and cen3tel 1000 cells was the up-regulation of several *MMP* genes and *Rnd3*, suggesting a transition towards a more mesenchymal phenotype in sphere cells compared to adherent cells. This observation is in agreement with several data of the literature indicating that, on the one hand, CSCs frequently show the acquisition of mesenchymal traits and, on the other hand, the induction of the epithelial-mesenchymal transition makes cells acquire a CSC phenotype^7^.

Several lines of evidence indicate that cholesterol metabolism is up-regulated in cancer^37^ and cancer stem cells^38–40^. Up-regulation of mevalonate/cholesterol metabolism genes is a main feature of cen3tel tumorspheres. Inhibition of HMGCR decreased cen3tel sphere formation, which was rescued by cholesterol, but not by mevalonate. This result is surprising, since mevalonate, being the product of the reaction catalysed by HMGCR, should allow the prosecution of the cholesterol biosynthetic pathway. A possible explanation for this observation is that, in the presence of simvastatin, the expression of enzymes downstream to HMGCR is not sufficient to allow an efficient cholesterol biosynthesis. Ginestier *et al*.^38^ reported that, in breast cancer cells, mevalonate but not cholesterol rescued sphere formation upon simvastatin treatment. However, it has to be pointed out that, in those experiments, cells grown in adhesion were exposed to the drug and one or the other metabolite and then seeded in sphere forming medium, while, in our experimental setting, cells were treated during sphere formation. Applying their experimental scheme, the same result was also obtained in cen3tel cells (data not shown). This can indicate that adherent cells require metabolites generated from pathways starting from mevalonate and collateral to cholesterol biosynthesis, while for cell growth in spheres, cholesterol is essential, thus being a possible target for CSC elimination.

Inflammation can have both a restraining and a promoting role in cancer^41,42^. *IL6* has been shown to be involved in cancer stem cell induction and maintenance^43–46^. Elevated levels of IL-13RA2 have been described in several cancers and have been implicated, for example, in metastatization and poor patients’ survival in colorectal cancer and ERα-negative breast cancer^47,48^. The IL-1β inflammatory cytokine encoded by *IL1B* plays a critical role in cancer progression, particularly in colorectal cancer^49^. *IL1B* is one of the most overexpressed genes in cen3tel 600 and 1000 sphere cells and, at the protein level, the IL-1β precursor form is undetectable in adherent cells and highly expressed in sphere cells. However, this protein does not appear to be processed and secreted in sphere cells. Further experiments are required to test whether IL-1β precursor can have a still unknown function in tumor cells and especially in CSCs.

Similarly, although interferon mediated responses can have an antitumor activity, recent evidence indicates that interferon signaling could play an important role in promoting tumor cell survival and mediating tumor growth; furthermore, a link has been shown between deregulation of the expression of interferon pathway related genes, neoplastic transformation and cancer stem cells^27,50^. In addition, pancreatic ductal carcinoma stem cells, enriched through the tumorsphere technique, show an increase in ISG15 expression and protein ISGylation and respond to ISG15 secreted by tumor associated macrophages enhancing the CSC phenotype^28^. It is also worth mentioning that ISG15 is overexpressed in human reprogrammed fibroblasts^51^ and in the stem cell fraction of mammospheres produced by normal human mammary glands^52^.

A strong transcriptional signature of tumorspheres is the overexpression *ISG15* and *STAT1*, with high levels of activated Stat1. Moreover, exogenous ISG15 increased sphere formation in both cen3tel cell populations. Thus, these observations confirm an involvement of ISG15 in CSC generation and suggest that ISG15 plays a crucial role in the maintenance of stemness properties of cen3tel tumorspheres.

Taken together, the results presented so far indicate that cen3tel spheres are characterized by gene expression changes that point towards stemness and tumorigenesis. *In vivo* tumorigenicity experiments have shown that sphere cells gave rise to tumor more quickly than adherent cells, but both cell populations induced tumors in NSG mice. This observation might be explained by the heterogeneity of sphere cells, by the segregation of the CSC phenotype during sphere cell proliferation in mice and also by a possible negative interaction of sphere cells with the mouse microenvironment. Nevertheless, it is also likely that different subpopulations, besides those selected by the tumorsphere approach, can support tumorigenicity in *in vitro* transformed cell lines.

In conclusion, this study shows that *in vitro* transformed cen3tel cells are heterogeneous and plastic populations, containing a subset of cells endowed with the capacity to grow in suspension as spherical aggregates in the absence of serum and in the presence of specific growth factors, a peculiar feature of CSCs derived from several tumors. These results confirm that differentiated cells undergoing oncogenic transformation can acquire CSC properties, supporting the hypothesis that bulk tumor cells are plastic entities able to switch between different phenotypes. However, cen3tel sphere cells are not the only tumorigenic population, suggesting that different subpopulations of transformed cells can support tumor development *in vivo*, thus adding a further layer of complexity to tumor heterogeneity.

## Methods

### The cen3tel cellular system

The cen3tel cellular system comprises cells at different stages along the way to transformation, well characterized at the cellular and molecular level^9–14^. Gene and miRNA expression profiles of cells at the different stages are available^13^. Cells around PD (population doubling) 30 after hTERT introduction maintained a behaviour similar to that of parental primary fibroblasts. Around PD 100, cen3tel cells started showing features of transformed cells, as the ability to grow in the absence of a solid surface, but were not able to induce tumors when injected into immunocompromised mice^9^, a feature that was achieved by cells around PD 160. Cen3tel cell tumorigenicity increased with further propagation in culture; in fact, inoculating 10^6^ cells under the skin of nude mice, the latency time for tumor formation decreased from about 1 month in cells around PD 160 to 8 days in cells around PD 600 and 2 days in cells around PD 1000. Tumorigenic cen3tel cells overexpress c-*MYC* and carry a mutation in *TP53* codon 160^10^, which has been used to confirm the identity of the cen3tel cells used in this work.

### Cell cultures and sphere formation assay

Cells were propagated in adherent culture conditions as previously described^11^. To perform sphere formation assays, cells were plated in Petri dishes (Corning, Tewksbury, MA, USA) at a concentration of about 10^4^ cells per ml of sphere-forming medium consisting in a 1:1 mixture of high glucose DMEM and Ham’s Nutrient Mixture F-12 (Euroclone, Siziano, Italy), supplemented with 20 ng/ml EGF and 20 ng/ml recombinant FGFb, 1X Insulin-Transferrin-Selenium (all from Life Technologies, Carlsbad, CA, USA), 4 mg/ml Bovine Serum Albumin (BSA, Sigma-Aldrich, Saint Louis, Missouri, USA), 2 mM glutamine (Euroclone), 50 U/ml penicillin, and 0.05 mg/ml streptomycin (Euroclone). After 6–7 days of growth, spheres were counted.

Spheres were dissociated with trypsin-EDTA and cells were used either for RNA or protein extraction, or replated either in sphere forming medium or in medium supplemented with serum to allow them to differentiate. To test single cell sphere formation, cells were seeded in 96 well plates (Corning) with a limiting dilution approach and wells containing a single cell were monitored for sphere formation. The frequency of sphere formation from single cells was calculated dividing the number of wells with a sphere by the total number of wells containing a single cell. To analyse the effect of ISG15 on sphere formation, 4 × 10^3^ cen3tel 600 or cen3tel 1000 cells/well were seeded in 24 well-plates (Corning) in sphere-forming medium supplemented with 100 ng/mL of ISG15 (Abcam, Cambridge, UK).

### *In vivo* tumorigenicity experiments and ethical approval

To test cell tumorigenic ability, cen3tel 600 or cen3tel 1000 cells were bilaterally injected into the leg muscle of NOD SCID Gamma (NSG) mice (Jackson Laboratory, Bar Harbor, Maine, USA) (from 4 to 6 mice for each cell sample). Mice were monitored for about 35 days assessing tumor appearance and measuring the tumor volume 1–2 times *per* week. Animal experiments were performed at the Humanitas Clinical and Research Center, Rozzano, Milan. Mice and tumors were used in compliance with national (4D.L.N.116, G.U., suppl. 40,18-2-1992) and international law and policies (EEC Council Directive 86/609,OJ L 358, 1, 12-12-1987; NIH Guide for the Care and Use of Laboratory Animals, US National Research Council, 1996). This investigation was approved by the Animal Care and Use Committee of the Humanitas Clinical and Research Center.

### Flow cytometric analysis

The Fixation/Permeabilization solution and Perm/Wash^TM^ Buffer (BD Biosciences, San Jose, CA, USA) were used. The anti-Sox2 antibody (S9072, Sigma-Aldrich) and the secondary Alexa Fluor^®^ 647-conjugated antibody (Thermo Fisher Scientific, Waltham, MA, USA) were diluted 1:200 and 1:500, respectively. The FACS Canto instrument and the FACS Diva and FlowJo software version 6.1.1 (BD Biosciences) were used for the analysis.

### RNA isolation, RT-quantitative PCR (RT-qPCR) and microarray analysis

RNA isolation and RT-qPCR were performed as described in Ostano *et al*.^13^. In Table S6, the list of genes analysed, together with the primers used, is presented.

For microarray analysis, RNA was extracted from two independent samples of adherently growing cells and 6 days spheres. Microarray analysis was done using the Agilent Whole Human Genome Microarray 4 × 44k glass slides (Agilent, Santa Clara, CA, USA). Probe preparation and hybridization were performed as previously described^11,13,53^, using a dye-swap analysis. After hybridization, slides were washed and scanned using the G2505C Agilent scanner.

Images were analysed using the Feature Extraction software v10.7 (Agilent). Raw data elaboration was carried out as previously described^13^. Separate channel analysis was performed in order to analyse two-color data in terms of individual channel intensities. The identification of differentially expressed genes in sphere cells versus adherent cells was performed as previously reported^13^, using R limma package. Relative changes in sphere cells versus adherent cells were expressed as base 2 logarithm of the ratio (log_2_FC) and only those transcripts with log_2_FC > 0.58 or <−0.58 and an adjusted p-value < 0.05 were considered as differentially expressed. Microarray data have been deposited in the NCBI database GEO (Gene Expression Omnibus), accession number GSE109787.

Unsupervised hierarchical clustering was performed on the global expression profiles of adherent and sphere cells. Euclidean distance as similarity metrics and Ward linkage method were used.

Gene Ontology (GO), pathway and gene set enrichment analysis on the list of differentially modulated genes both in cen3tel 600 and 1000 sphere cells were performed using David (http://david.abcc.ncifcrf.gov/), the Panther Classification System (http://www.pantherdb.org/index.jsp) and GSEA (http://software.broadinstitute.org/gsea/index.jsp)^54^, respectively. p < 0.05 was used for the identification of overrepresented processes.

### Western blot analysis

Whole protein extracts were prepared using the RIPA lysis buffer as described in Belgiovine *et al*.^11^. The primary antibodies used are listed in Table S7. HRP-conjugated secondary antibodies were from Jackson ImmunoResearch (West Grove, PA, USA). To assess for the presence of ISG15 (MW 17 KDa) in the culture medium (CM), about 4 mL of medium were collected from a 7-day sphere culture (about 1500 spheres/ml) and concentrated about 70 folds. 20 µl of the concentrated culture medium were analyzed by western blotting using an antibody against ISG15 (Table S7).

### Statistical analysis

Results were presented as the mean ± standard deviation (SD) and analyzed using Student’s *t*-test. *P*-values lower than 0.05, 0.01 or 0.005 were considered significant.

## Electronic supplementary material

Supplementary information

Table S2

Table S3

Table S4

Table S5

## Data Availability

The datasets generated during the current study are available in the NCBI database GEO (Gene Expression Omnibus), accession number GSE109787.
