# Supplementary material for: Cells with stemness features are generated from in vitro transformed human fibroblasts
Source: Sci Rep. 2018 Sep 14;8:13838. doi: 10.1038/s41598-018-32197-5 (PMC6138721; doi:10.1038/s41598-018-32197-5)

Cells with stemness features are generated from in vitro transformed human fibroblasts

Bartolo Bono, Paola Ostano, Martina Peritore, Ilaria Gregnanin, Cristina Belgiovine, Manuela Liguori, Paola Allavena, Giovanna Chiorino, Ilaria Chiodi, Chiara Mondello

Supplementary Information

Supplementary Tables

Table S1. **Sphere formation frequency in cen3tel 600 and derived clones**

**Cells**	**Spheres (%)**
Cen3tel600	7.7
Clone 3	15.1
Clone 5	11.3
Clone 8	7.4
Clone 11	22.1
Clone 19	8.0
Clone 20	6.0
Clone 21	17.3
Clone 23	12.6

Clones were grown for about 23 PDs before plating in sphere forming medium.

Table S6. **List of the qPCR primers**

A QuantiTect Primer Assays

Gene Symbol	Primer Name	Amplicon length (bp)
GNL3	Hs_GNL3_1_SG	141
GUSB	Hs_GUSB_1_SG	96
HMGCR	Hs_HMGCR_1_SG	87
HMGCS1	Hs_HMGCS1_1_SG	123
IFI6	Hs_IFI6_1_SG	108
IL13RA2	Hs_IL13RA2_1_SG	82
MMP1	Hs_MMP1_1_SG	103
MVK	Hs_MVK_1_SG	124
c-MYC	Hs_MYC_1_SG	129
NANOG	Hs_NANOG_2_SG	164
NOTCH1	Hs_NOTCH1_2_SG	92
POU5F1	Hs_POU5F1_1_SG	77
SOX2	Hs_SOX2_1_SG	64

B Custom primers

Gene Symbol	Primer sequence (5' - 3')	Amplicon length (bp)
MMP7	Fw:ATGGGGAACTGCTGACATCAT	153
	Rev:CCAGCGTTCATCCTCATCGAA	
MMP14	Fw:CTAAGACCTTGGGAGGAAAAC	192
	Rev:AAGCCCCATCCAAGGCTAACA	

Table S7. List of the antibodies used for western blotting

Target	Manufacturer	Code	Dilution
BCL2A1	Abcam	ab33862	1:500
Cleaved caspase-3	Enzo Life Science	ALX-210-807-C100	1:500
Cleaved caspase-9	Cell Signaling	#9505	1:500
c-Myc	Abcam	ab32072	1:10000
ISG15	Santa Cruz Biotechnology	A-4	1:500
IL-1β	R&D Systems	MAB601	1:500
Notch1	Abcam	ab52627	1:1000
Nucleostemin	Bethyl	A300-600A	1:2000
PARP-1	Abcam	ab191217	1:1000
poly(ADP-ribose)	Enzo Life Sciences	ALX-804-220	1:1000
RhoE/Rnd3	Sigma-Aldrich	R6153	1:500
Stat1 p84/p91	Santa Cruz Biotechnology	C-136	1:500
Phospho-Stat1 (Tyr701)	Cell Signaling	D4A7	1:1000
γ-Tubulin	Sigma-Aldrich	T6557	1:5000

Antibodies were used diluted in 3% skimmed milk/1X TBS or in 5% BSA/1X TBS 0.1% Tween 20.

Supplementary Figures

Supplementary figure legends

Fig. S1 Validation of microarray results by RT-qPCR. Expression levels of *HMGCS1*, *HMGCR* and *MVK* (a), *IL13RA2* (b) and *IFI6* (c) in sphere cells are indicated as Log_2FC relative to the corresponding adherent cells and are the mean of the results of three independent experiments.

Fig. S2 Original images of western blot images. Area surrounded by black lines are shown in the corresponding figure panels in the manuscript**.**

Fig. S1

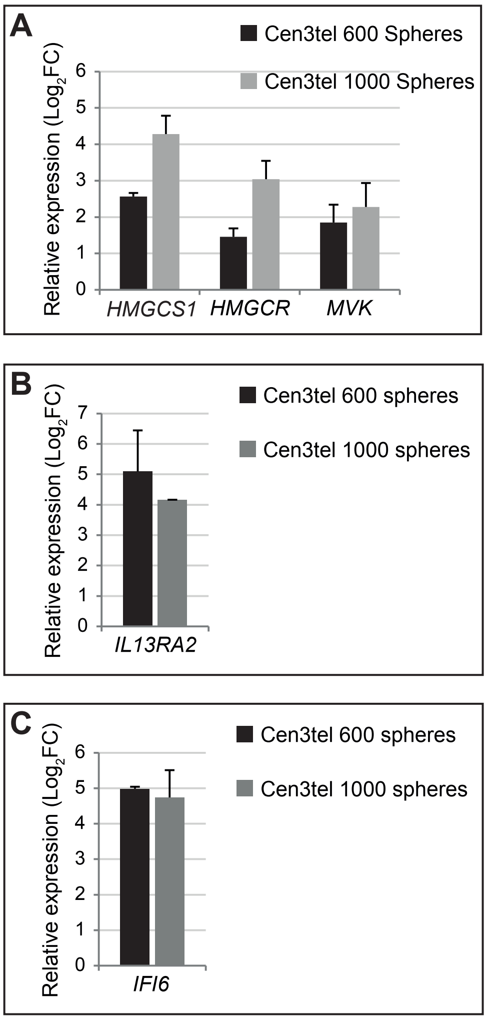
A
Cen3tel 600 Spheres
Cen3tel 1000 Spheres
Relative expression (Log2FC)
6
5
4
3
2
1
0
HMGCS1
HMGCR
MVK
B
Cen3tel 600 spheres
Cen3tel 1000 spheres
Relative expression (Log2FC)
7
6
5
4
3
2
1
0
IL13RA2
C
Cen3tel 600 spheres
Cen3tel 1000 spheres
Relative expression (Log2FC)
6
5
4
3
2
1
0
IFI6

Fig. S2 Uncropped western blotting images

Figure 2 Panel a

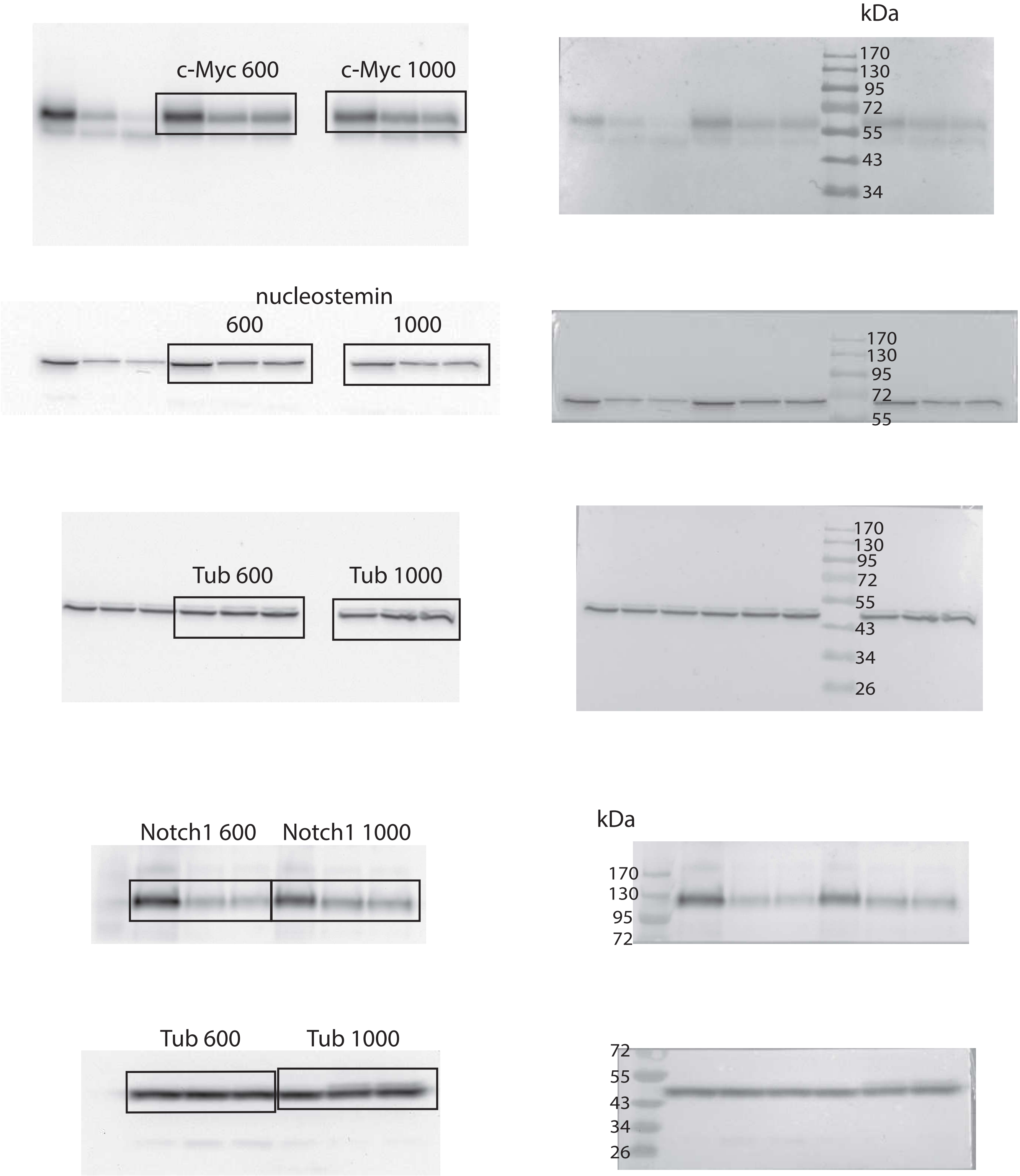

Figure 2 Panel c cen3tel 600

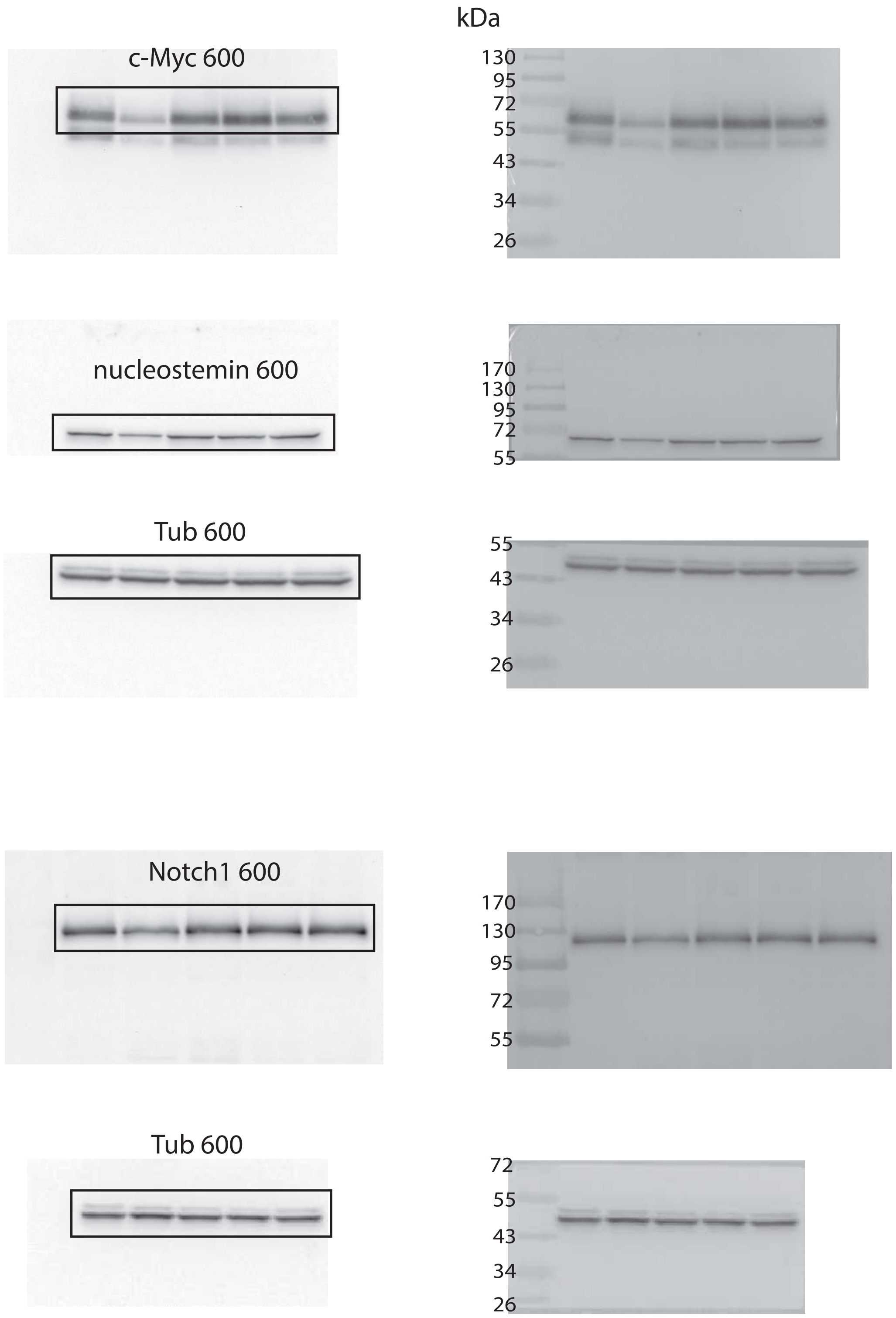

Figure 2 Panel c cen3tel 1000

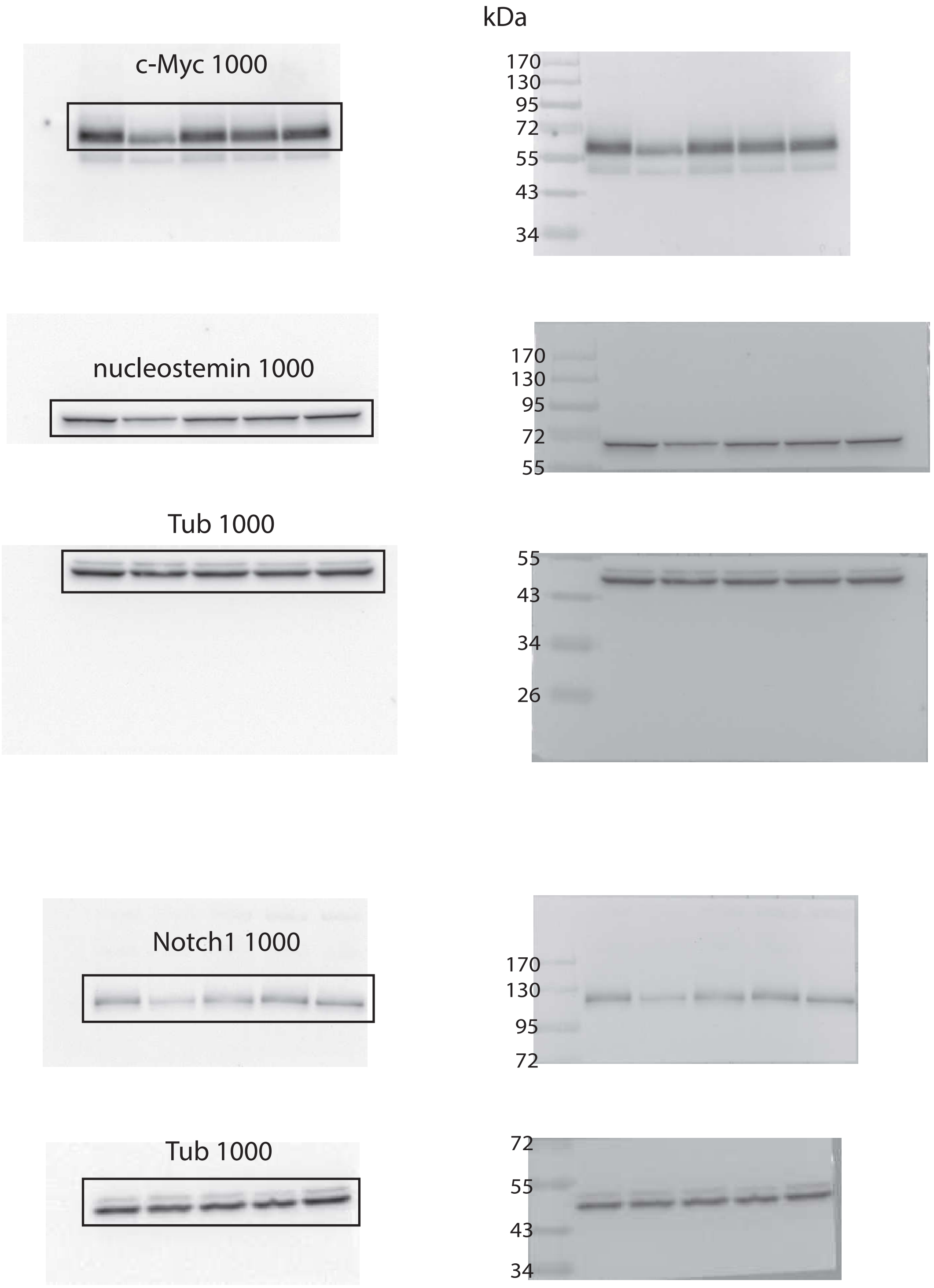

Figure 2 Panel e

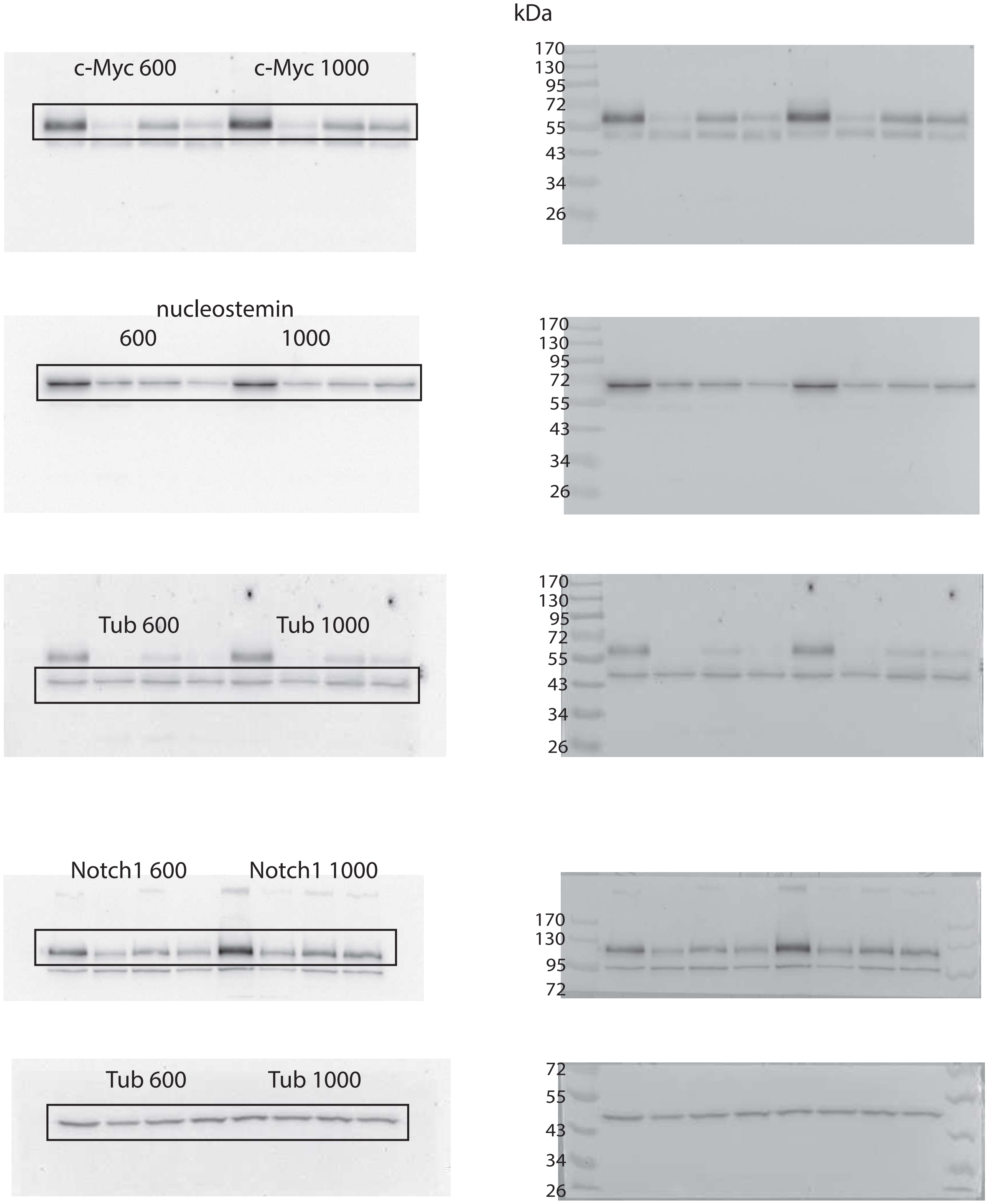

Figure 3 Panel a

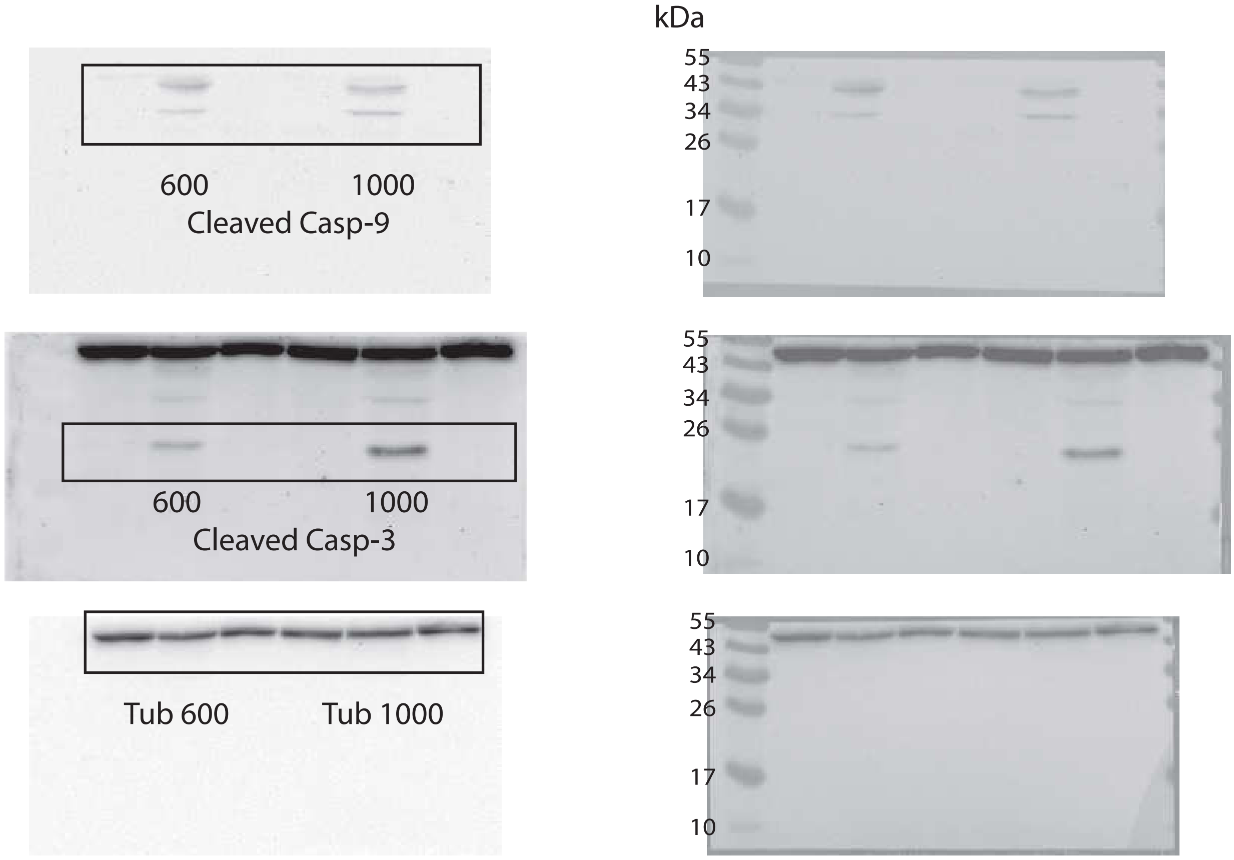

Figure 3 Panel b

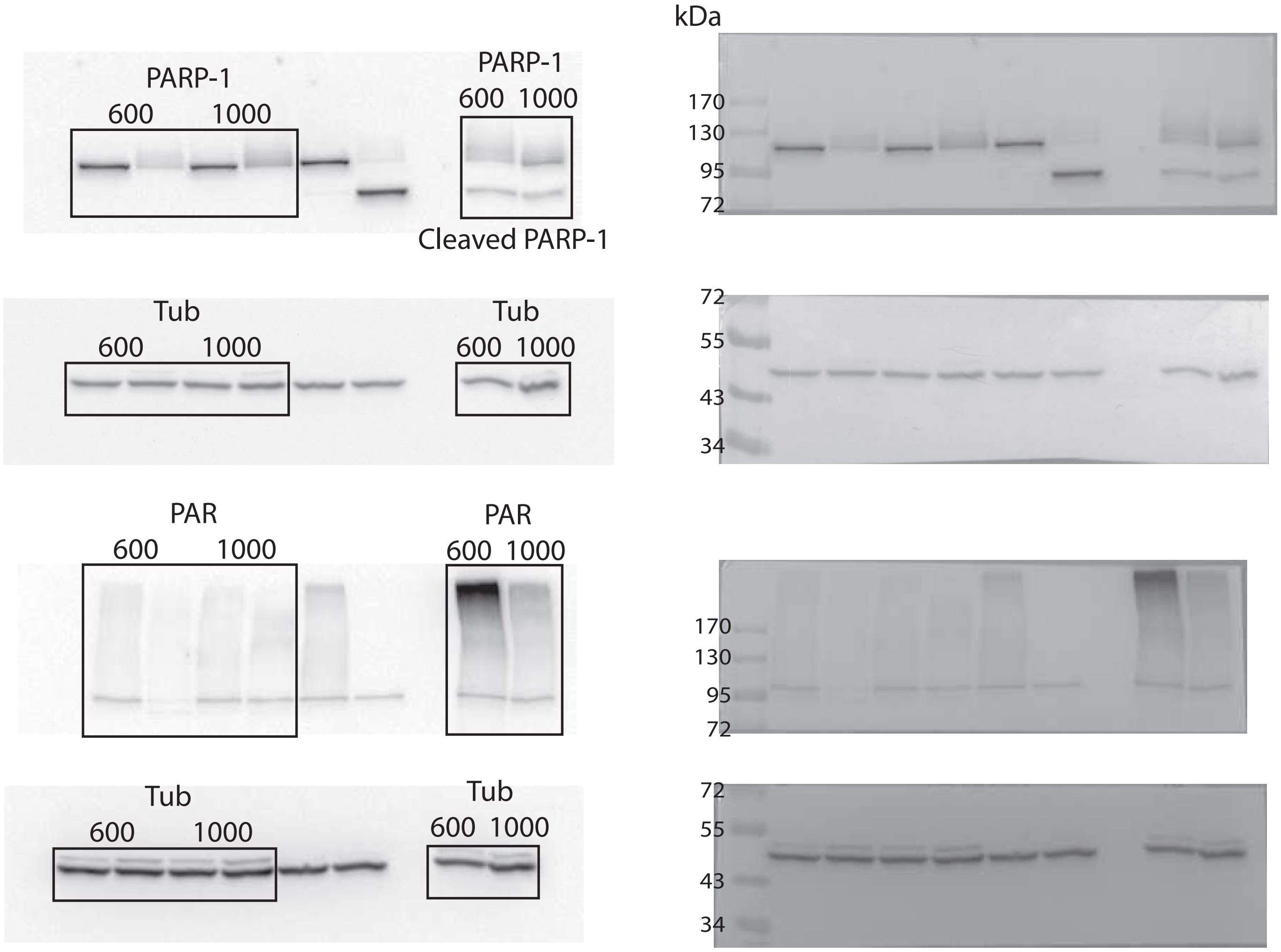

Figure 4 Panel e

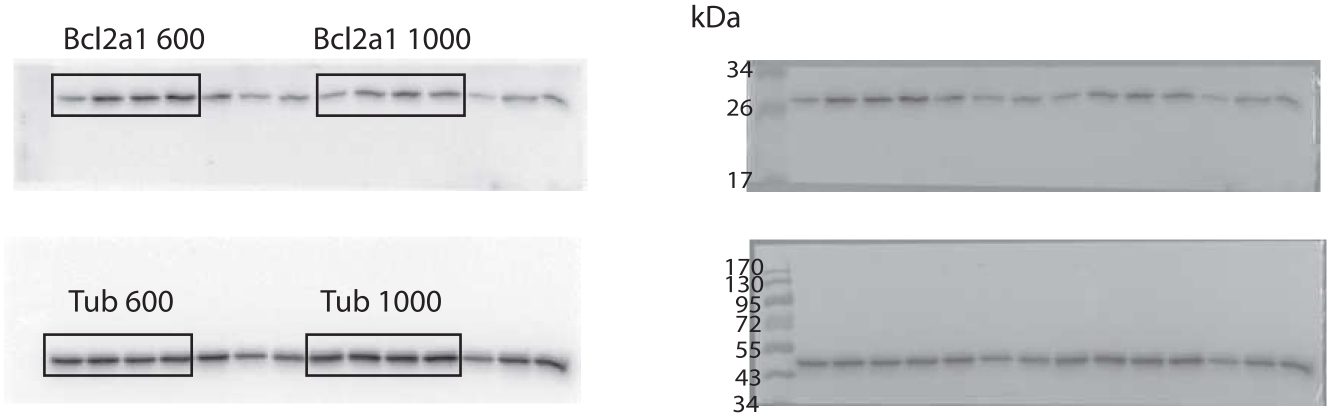

Figure 4 Panel g

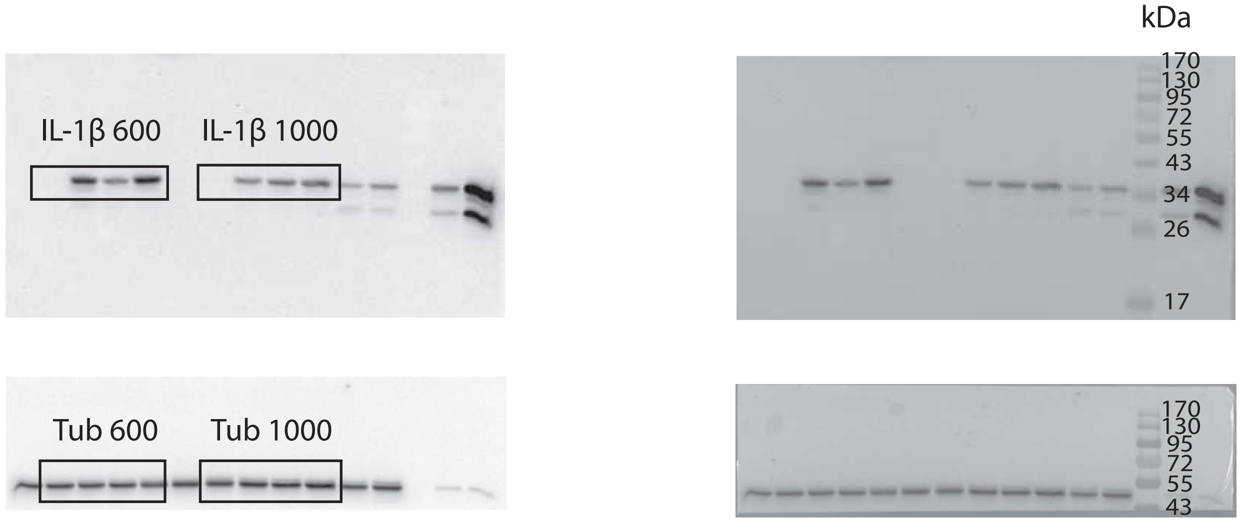

Figure 5 Panel a

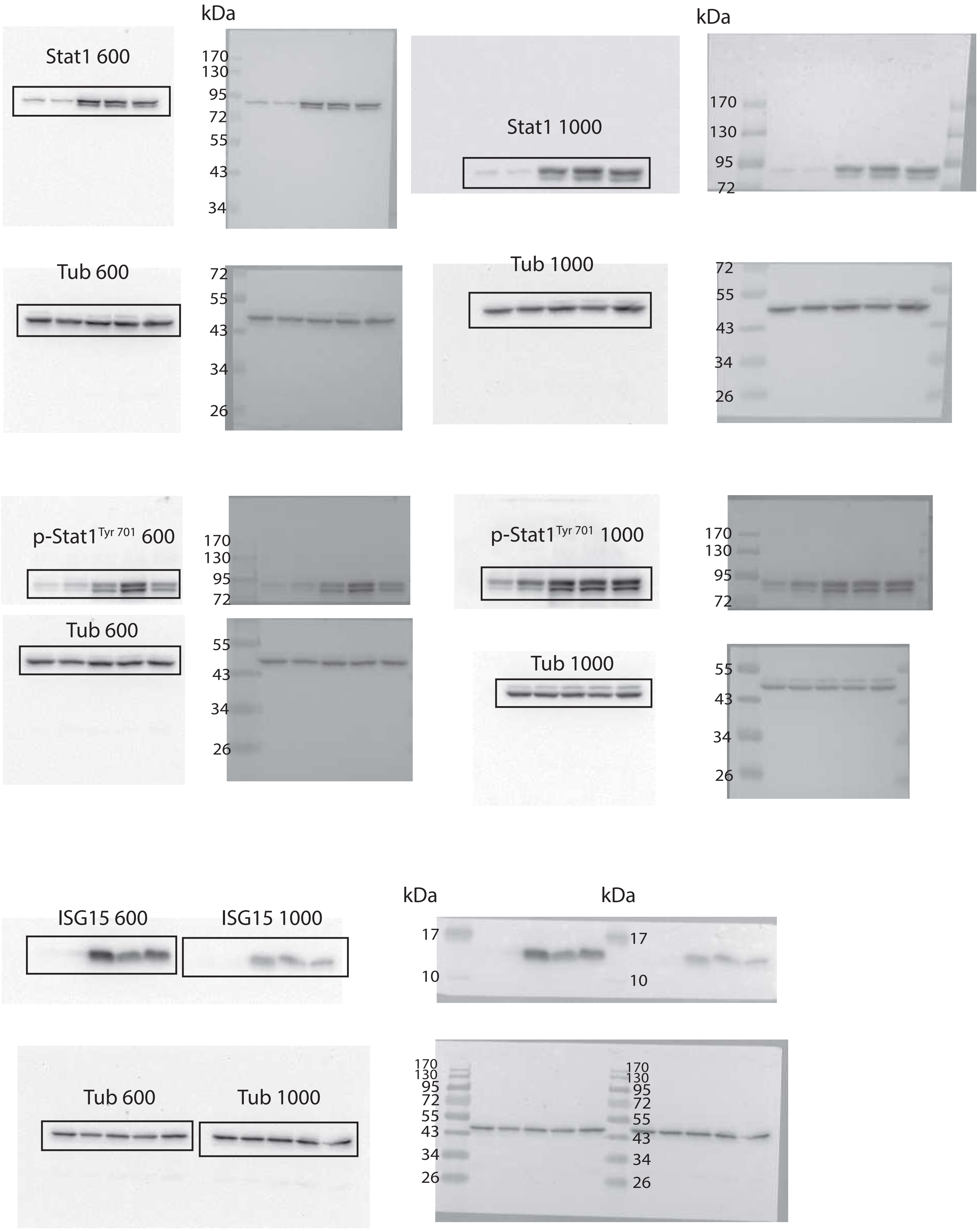

Figure 5 Panel a ISGylation

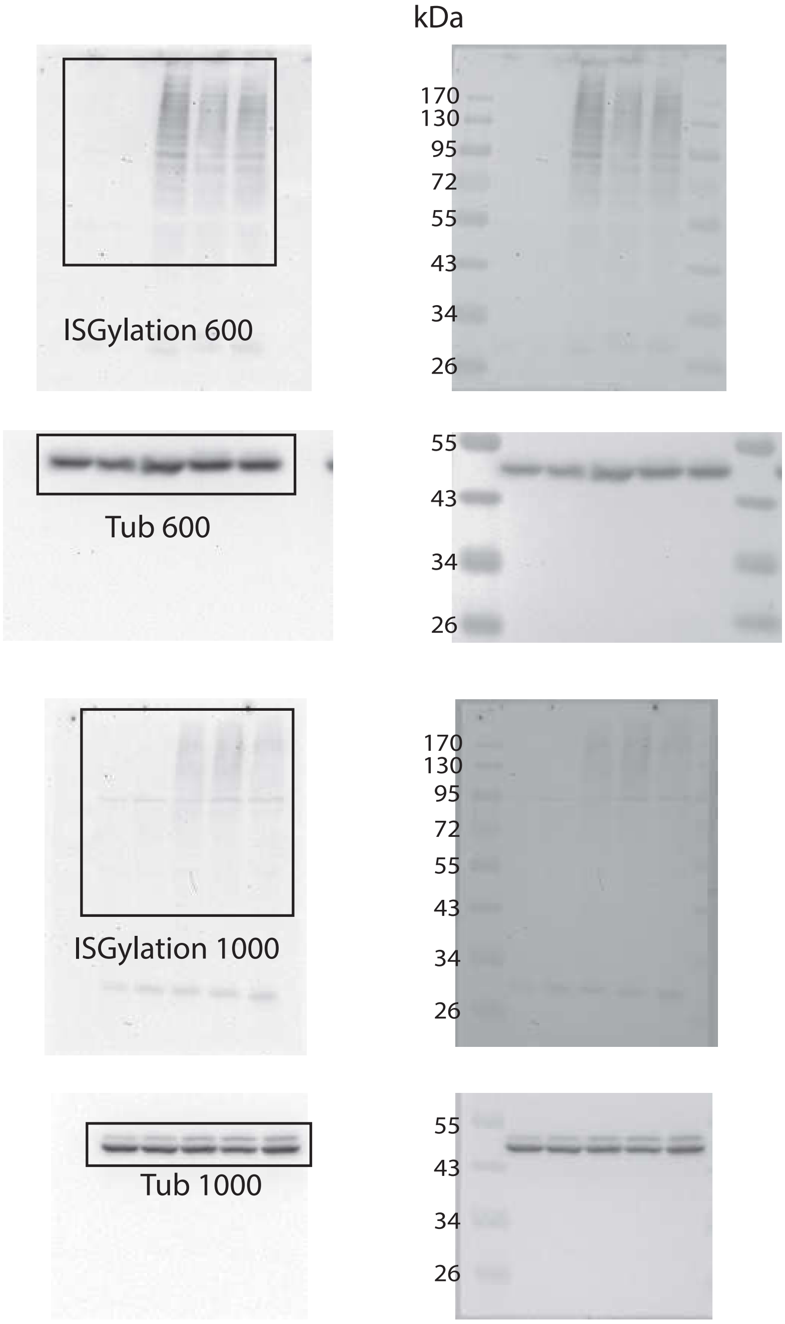

Figure 5 Panel b ISG15 culture medium

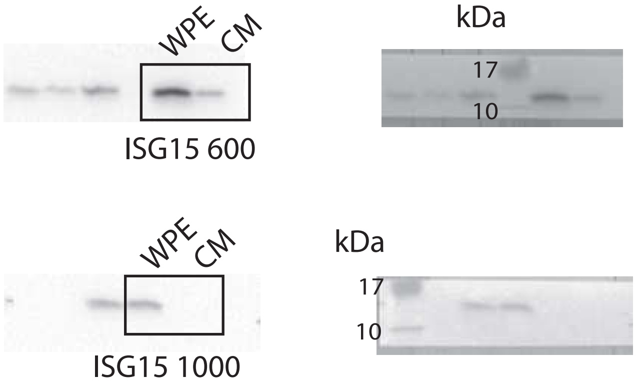

Figure 6 Panel b

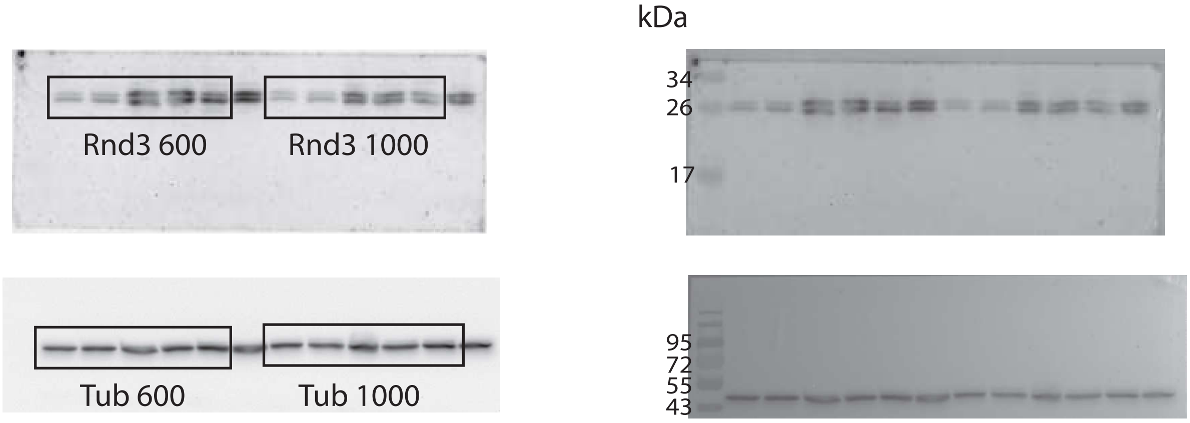

Supplement: Supplementary file 1 — Supplementary information [file 41598_2018_32197_MOESM1_ESM.pdf]
